# Spatio-temporal expression patterns of anandamide-binding receptors in rat implantation sites: evidence for a role of the endocannabinoid system during the period of placental development

**DOI:** 10.1186/1477-7827-7-121

**Published:** 2009-10-27

**Authors:** Bruno M Fonseca, Georgina Correia-da-Silva, Anthony H Taylor, Justin C Konje, Stephen C Bell, Natércia A Teixeira

**Affiliations:** 1 Serviço de Bioquímica, Faculdade de Farmácia, Universidade do Porto, Porto, Portugal; 2 Instituto de Biologia Molecular e Celular da Universidade do Porto (IBMC), Porto, Portugal; 3 Endocannabinoid Research Group, Reproductive Sciences Section, Department of Cancer Studies and Molecular Medicine, University of Leicester, Leicester, UK

## Abstract

**Background:**

Although there is growing evidence that endocannabinoids play a critical role in early pregnancy, there are no studies describing the possible targets for this system after implantation. The endometrial stroma, which undergoes extensive proliferation and differentiation giving rise to the decidua and the trophoblast cells that invade after the initial stages of implantation, are potential targets. Since high anandamide (AEA) levels, the main endocannabinoid, are detrimental to implantation and in order to gain insight into the role of the endocannabinoid system in the development of the fetoplacental unit, the spatio-temporal pattern of expression of the anandamide-binding receptors, CB1, CB2 and the vanilloid receptor (TRPV1), were investigated by quantitative RT-PCR, western blot and immunohistochemistry.

**Methods:**

Rat uterine maternal tissues from different days of pregnancy were used to investigate the expression of CB1, CB2 and vanilloid receptors by quantitative RT-PCR, western blot and immunohistochemistry.

**Results:**

The data indicate that all the three receptors were expressed in decidualized cells and placenta. Interestingly, CB1 and CB2 were also expressed in smooth muscle cells of maternal blood vessels and in endovascular trophoblast cells, whereas TRPV1 was mainly expressed in uterine natural killer (uNK) cells and in the longitudinal muscle layer throughout pregnancy. In all tissues, CB2 protein was present at a lower level than CB1.

**Conclusion:**

These observations support a role for the endocannabinoid system during the period of decidualization and placental development.

## Background

Endocannabinoids are increasingly being recognised as regulators of key cell-signalling pathways in female reproduction. These long-chain polyunsaturated fatty acid lipid mediators mimic many of the effects of Δ^9^-tetrahydrocannabinol (THC), the principal psychoactive compound of marijuana, which has been reported to have adverse effects on reproductive functions, including retarded embryo development, fetal loss and pregnancy failure [1,2]. *N*-arachidonoylethanolamine (anandamide, AEA) and 2-arachidonylglycerol (2-AG) are the two main endocannabinoids that have been characterized [3,4]. They are not stored but produced by most tissues on demand via a variety of stimuli that act on cannabinoid receptors, of which CB1 and CB2 are the two main subtypes. CB1 and CB2 are seven transmembrane G protein-coupled receptors which on activation have different biological effects that are cell-type specific and context-dependent [5,6]. It has been shown that anandamide evokes a wide spectrum of physiological actions, such as analgesia, vasodilatation, cell proliferation and cell death, through these receptors [7].

CB1 receptors were originally demonstrated in the central nervous system, and CB2 was originally found to be expressed in the spleen and immune cells [8,9]. Both receptors are now known to be expressed at low levels in tissues of the reproductive tract [10] and have been shown to be functional in these tissues.

Anandamide has also been shown to have non CB1 and non CB2-dependent effects suggesting the existence of a CB3 receptor [11] and evidence exists that anandamide can also bind to other receptors that are not exclusively associated with cannabinoids [12]. Indeed, it has been shown that anandamide binds to and activates the transient receptor potential vanilloid 1 receptor (TRPV1 or VR1), which is characterized as a ligand-gated non selective cationic channel [13,14].

In the human, successful implantation and pregnancy progression is characterized by low plasma levels of AEA, while in labour the level of this endocannabinoid dramatically increases [15]. Additionally, high levels of plasma AEA in early pregnancy [16] or low fatty acid amide hydrolase (FAAH) levels and activities [17] have been shown to be associated with miscarriage. Low levels of AEA have also been reported in the late luteal phase of the menstrual cycle, coincident with the window of uterine receptivity for implantation, supporting the importance of the endocannabinoid system in early pregnancy [18]. In fact, endocannabinoid signalling is a critical pathway in embryo-uterine cross-talk for successful blastocyst activation and implantation [19]. Low levels of AEA in the receptive uterus and activated CB1 in blastocysts are beneficial for implantation, whereas higher AEA levels are detrimental to this process [20-22]. This dose-dependent effect of AEA suggests a biphasic role for AEA in implantation and is further supported by findings showing that a very narrow range of AEA concentrations regulate blastocyst activation and implantation by differentially modulating MAP kinase signalling and Ca^2+ ^channel activity via the CB1 receptor [19,23].

Following implantation, the uterus undergoes extensive tissue remodelling, in order to achieve a successful pregnancy. In rodents, decidualization, which involves proliferation and differentiation of the endometrial stromal cells, starts in the vicinity of the implanting blastocyst giving rise to the antimesometrial decidua [24]. Thereafter, decidualization expands to the opposite side of the uterus (the mesometrial pole) to form the mesometrial decidua. Development of this tissue is also associated with the appearance of a third type of cell population, the uterine natural killer cells (uNK), which have been implicated in both successful pregnancy and pregnancy problems [25]. After full development, both decidual zones regress by programmed cell death; however, not simultaneously, suggesting that paracrine or autocrine mechanisms might control apoptosis in specific regions of the decidua [26,27]. These changes are considered to be crucial for successful pregnancy but little is known about the molecular mechanisms involved in these remodelling processes.

Since the endocannabinoid system has been suggested as one of the key cytokine signalling pathways regulating blastocyst maturation, oviductal transport, implantation and maintenance of early pregnancy, we hypothesised that important critical changes in this system in the maternal component of the fetal-maternal interface would occur during pregnancy. While endocannabinoid signalling is clearly critical to early pregnancy events, effects in the post implantation period and implications in pregnancy outcome remain unknown. Moreover, we have recently shown the expression of both cannabinoid receptors, as well as, TRPV1 in mesometrial decidual cells at day 10 of pregnancy. In addition, we also demonstrated the involvement of CB1 receptor in decidual cell death [28]. However, it was not been described the presence of these molecules after this day of pregnancy. Therefore, in order to clarify the role of endocannabinoids it was investigated the spatio-temporal pattern of expression of the cannabinoid receptors CB1 and CB2, as well as, of the related receptor, TRPV1 throughout gestation.

## Methods

### Animals and tissue preparation

Nulliparous Wistar rats weighing 200-250 g (Charles River Laboratories, Barcelona, Spain) were kept under standard 12 h/12 h light/dark conditions in the laboratory animal care facility of our institution. All procedures involving animals were conducted in accordance with the guidelines of the Ethics Committee of the Institute of Molecular and Cellular Biology, Oporto University. Female rats were mated with males and the day on which spermatozoa were found in the morning vaginal smear was designated day 1 of pregnancy. On days 8, 10, 12, 14, 16 and 19 of pregnancy, animals were sacrificed by cervical dislocation, and uteri were collected, fixed in 10% (v/v) formal saline for 24 to 48 h and processed for routine paraffin histology. The paraffin block was orientated to enable the implantation sites to be sectioned transversally. Serial sections (4 μm) through each implantation site in the area containing the embryo were placed onto slides coated with aminopropyl-triethoxysilane (Sigma Chemical Co, St Louis, USA). Slides were dewaxed, hydrated and stained with standard hematoxylin and eosin to determine general tissue morphology and to identify the different cell types present.

For the preparation of tissue homogenates, brain, spleen and uterine horns, from day 10 until day 19 of pregnancy, were dissected out and the maternal tissues separated from placenta and homogenized with a Potter homogenizer in a buffer consisting of 20 mM Hepes buffer, 2 mM EDTA, 10 mM KCl, 1.5 mM MgCl_2 _(1:2) 1 mM phenylmethylsulphonylfluoride (PMSF) and aprotinin (1:100). The homogenates were centrifuged at 700 *g *for 10 min at 4°C to remove cell membranes and nuclei. The supernatants were then centrifuged at 12000 *g *for 30 min at 4°C and the supernatants stored at -80°C. The amount of protein was measured by the Bradford assay kit (Bio-Rad, Laboratories Melville, NY, USA).

For RT-PCR analyses, 100 mg of tissue was homogenised directly in homogenisation buffer (Qiagen RNAEasy kit; Qiagen, Hilden, Germany) and the RNA purified through Qiagen columns according to the manufacturer's instructions. The RNA was eluted in 30 μl of RNAse-free water and stored at -80°C for later RT-PCR analysis.

### Quantitative RT-PCR analysis

RNA was quantified spectrophometrically at 260 and 280 nm and RNA quality assessed using ethidium-stained agarose gels. RNA with a 260/280 ratio of 1.8 and above was reverse-transcribed using avian myelomablastosis reverse transcriptase (Promega Corp. Southampton, UK) as previously described [29]. For quantitative PCR, gene specific primers (Table 1) were used at 10 pmol/μl in a SYBR green system (Roche Diagnostics, Lewes, UK) with 1 μl of cDNA as template in a Roche Lightcycler 1.2. The PCR conditions in all cases started with a denaturation step at 95°C for 10 min and followed by up to 50 cycles of denaturation, annealing and primer extension (Table 1). Standard curves of diluted cDNA pools were constructed for each gene target and the expression levels corrected for the levels of rat β-actin using the 2^-ΔΔCt ^method [30] and normalised to the ratio produced from the day 10 of pregnancy sample.

**Table 1:** Primer sequences, gene accession number and Q-PCR conditions.

**mRNA target**	**GenBank accession No.**	**Primers**	**Conditions**	**Ref.**
*β-actin*			95°C, 15 sec	Cintado *et al*., 2001
	NM_031144	Sense 5'-CCTAGCACCATGAAGATCAA-3'	60°C, 20 sec	
		Antisense 5'-TTTCTGCGCAAGTTAGGTTTT-3'	72°C, 30 sec	
				
*Cb1*	NM_012784		95°C, 20 sec	Porcella *et al*., 1998
		Sense 5'-CATCATCATCCACACGTCAG-3'	60°C, 30 sec	
		Antisense 5'-ATGCTGTTGTCTAGAGGCTG-3'	72°C, 20 sec	
				
*Cb2*	NM_020543		95°C, 20 sec	Porcella *et al*., 1998
		Sense 5'-TTTCCCACTGATCCCTAACG-3'	60°C, 30 sec	
		Antisense 5'-AGTTAACAAGGCACAGCATG-3'	72°C, 20 sec	
				
*Trpv1*	AF237067		95°C, 25 sec	Dvorakova *et al*., 2001
		Sense 5'-TGGAACAACGGGCTAGCTTA-3'	58°C, 30 sec	
		Antisense 5'-TCCTCATAAGGGCAGTCCAG-3'	72°C, 25 sec	

CB1, cannabinoid receptor 1; CB2, cannabinoid receptor 2; TRPV1, transient receptor potential vanilloid 1 receptor

### Western blot analysis

Homogenized samples were resolved by electrophoresis on a 10% SDS-polyacrylamide gel. The separated proteins were transferred onto a nitrocellulose membrane in 25 mM Tris-HCl, 250 mM glycine, 20% methanol (v/v), pH 8.3, for 2 h at 200 V. The membranes were incubated with a 1:100 dilution of polyclonal antibodies against CB1 (sc-20754), CB2 (sc-25494) or TRPV1 (sc-12498) from Santa Cruz Biotechnology (Santa Cruz, CA, USA) in blocking solution overnight at 4°C. Membranes were exposed to X-ray film (Kodak XAR; Eastman Kodak, Rochester, NY, USA) and bands were visualized by chemiluminescence (Super Signal West Pico; Pierce, Rockford, USA). The signal intensity was quantified by densitometry (BIO-PROFIL Bio-1D++, Vilber Lourmat, France). Homogenates of brain and spleen were used as positive controls for CB1, TRPV1 and CB2, respectively. Membranes were then stripped and incubated with anti-β-tubulin (1:500) (Santa Cruz, CA, USA) antibody to control loading variations. A total of four animals were used for all the experiments.

### Immunohistochemistry

Expression of CB1, CB2, and TRPV1 was analyzed using an avidin-biotin alkaline phosphatase complex immunohistochemical technique (Vectastain ABC kit, Vector Laboratories, CA, USA). After dewaxing, rehydration and blocking of non-specific binding sites, slides were incubated, overnight with the primary antibodies rabbit anti-CB1 and anti-CB2 (1:100) and goat anti-TRPV1 (1:100) at 4°C. After washing with PBS they were incubated with diluted biotinylated secondary antibody for 30 min, followed by incubation with Vectastain ABC-AP reagent, as recommended in the kit instructions. The reaction was developed by incubation with Sigma Fast Red tablets (Sigma Chemical Co, St Louis, USA). Negative controls were performed with the inclusion of rabbit or goat IgG instead of the CB1/CB2 or TRPV1 primary antibodies. The slides were counterstained with Mayer's hematoxylin solution (Sigma Diagnostics, St Louis, USA) and mounted in Aquamount improved medium (BDH Laboratory Supplies, Poole, England). In each experimental immunohistochemical run, sections from all days of pregnancy (days 8 to 19) were included and a total of three implantation sites from five different animals were used for all the experiments.

### Statistical analysis

Statistical analysis was performed using one-way ANOVA, followed by the Bonferroni post-hoc test to make pairwise comparisons of individual means (GraphPad PRISM version 4.0, GraphPad Software, Inc., San Diego, CA, USA) when significance was indicated. All numerical data are expressed as mean ± SEM and differences were considered to be statistically significant at *p *< 0.05.

## Results

### CB1, CB2 and TRPV1 mRNA levels

**Figure 1:**
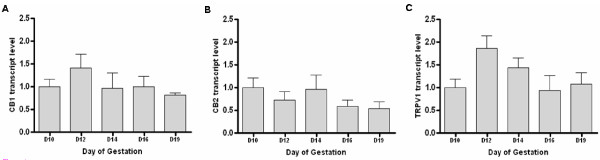
**Anandamide binding receptor transcript levels during pregnancy**. The levels of transcripts for (A) CB1, (B) CB2 and (C) TRPV1 were measured by Q-PCR and normalised against rat β-actin levels. The data were then divided by the mean value obtained on Day 10 and presented as mean ± SEM; n = 4.

Figure 1 shows the relative levels of transcripts for CB1, CB2 and TRPV1 in the uterine maternal tissues from day 10 to day 19 of pregnancy. While for CB1 and TPVR1 a trend to increase on day 12 was detected, for CB2 this tendency was observed on day 10 and 14. The pattern of transcript expression for CB1 and TRPV1 was similar. However, the differences in transcript levels for all three receptors were not significantly different between the days of gestation.

### Western blot analysis

**Figure 2:**
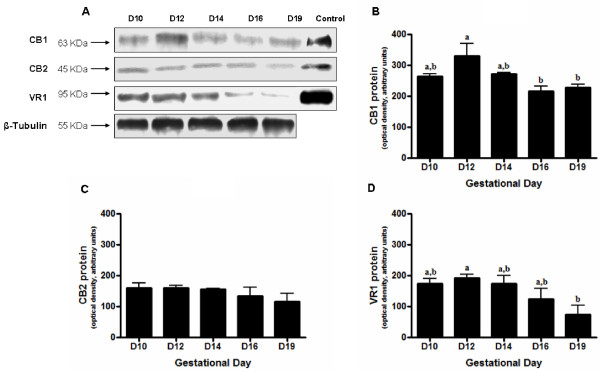
**Anandamide-binding receptor protein levels in mesometrial decidua during pregnancy**. (A) Representative western blots for CB1, CB2, TRPV1 and β-tubulin of protein extracts from mesometrial decidua taken on days 10, 12, 14, 16 and 19 of pregnancy. Brain homogenates were used as positive controls (control) for CB1, TRPV1 and spleen homogenates for CB2. β-tubulin was used as a loading control. (B) Densitometric analysis of CB1, CB2 (C) and TRPV1 (D) protein levels. The different protein levels were normalized to the level of β-tubulin. Bar graphs show the mean of experiments carried out with four different animals. Results are depicted as means ± SEM. Data labelled with different letters are significantly different from each other.

The relative abundance of CB1, CB2 and TRPV1 protein in the mesometrial decidua throughout pregnancy was also evaluated by immunoblotting (Figure 2). In contrast to the transcript levels, there were significant differences in the protein levels of the receptors between days of gestation. CB1 protein levels on day 12 were significantly (*p *< 0.05) higher than those on days 16 and 19 of pregnancy (Figure 2A, B). CB2 was also expressed in the mesometrial decidua throughout the period in study, however, the absolute protein levels appeared to be less than that shown by CB1. In contrast to CB1, there were no statistically significant differences in the protein levels for CB2 (Figure 2A, C). TRPV1 was also present in all days of pregnancy, and after day 14 declined towards the end of gestation, being the protein levels significantly (p < 0.05) elevated on day 12 when compared to the end of gestation (Figure 2A, D).

### Expression pattern of CB1, CB2 and TRPV1 receptor - Days 8 to 10 of pregnancy

Since only significant variations between days of pregnancy were found at the protein level, the spatio-temporal pattern of CB1, CB2 and TRPV1 expression was investigated by immunohistochemistry.

**Figure 3:**
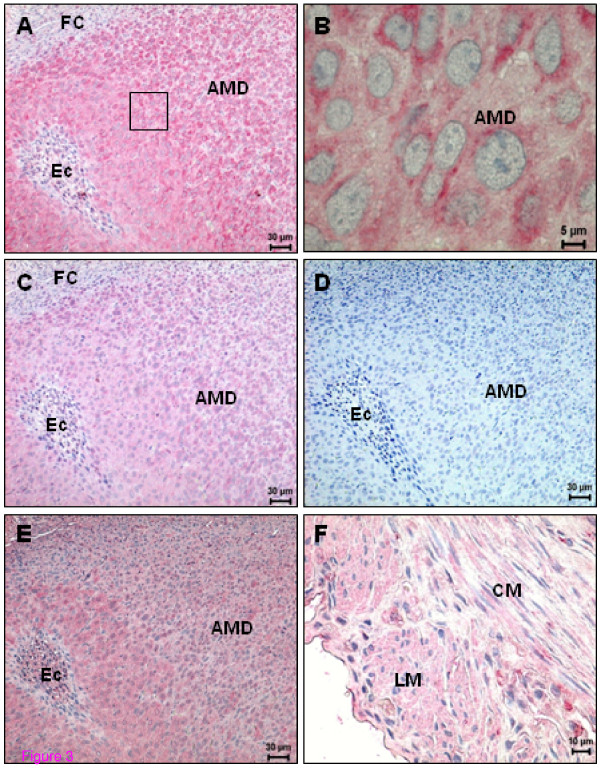
**Immunohistochemical demonstration of anandamide-binding receptor expression in the rat uterus on day 8 of pregnancy**. (A) CB1 expression is evenly distributed in the antimesometrial decidua; (B) Higher magnification of the square indicated in (A); (C) CB2 expression in decidual cells of antimesometrium, note that CB2 staining appears to be less intense than CB1; (D) A negative control for CB2 of a consecutive section of (C); (E) TRPV1 expression; note a higher expression in the cells close to the embryo; (F) TRPV1 expression in longitudinal and circular muscle layers. AMD - Antimesometrial decidua; CM - Circular muscle layer; Ec - Embryonic cells; FC - Fibrinoid capsule; LM - Longitudinal muscle layer.

Decidualized cells of the antimesometrium exhibited a high expression for CB1 receptor compared with that for CB2 (Figure 3A, B, C). Although TRPV1 was positive in the antimesometrial decidua on days 8 and 10, the expression was more intense in the vicinity of the embryo (Figure 3E). In the antimesometrial pole, near the circular muscle coat, the fibrinoid capsula, a layer of closely packed cells adjacent to the undifferentiated endometrium [31], was negative for all three receptors (Figure 3A, C).

**Figure 4:**
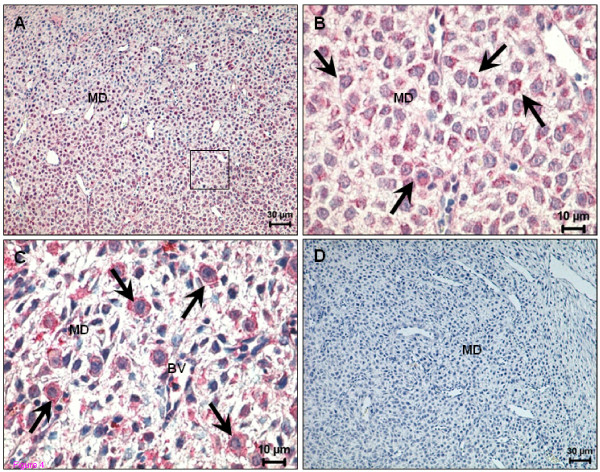
**Immunohistochemical demonstration of anandamide-binding receptors expression in uterus on day 10 of pregnancy**. (A) CB1 expression in the decidualized cells in the mesometrium pole; (B) Higher magnification of the square indicated in (A) showing positive staining in precursors of uNK cells (arrows); (C) TRPV1 expression in the mesometrial pole in uNK cells (arrows); (D) A negative control for CB1. BV - Blood vessel; MD - Mesometrial decidua.

The mesometrial decidual cells started to show immunopositivity for both cannabinoid receptors by day 10 (Figure 4A, B), although again CB2 staining was less intense when compared to that for CB1. In addition, a few small round cells, the precursors of uterine NK cells, which appear later in the mesometrial triangle and in the mesometrial decidua, expressed CB1 (Figure 4B). The smooth muscle cells of blood vessels exhibited immunopositivity for CB1 and CB2 (data not shown). Only a few decidual cells of the mesometrium and the uNK cells began to express TRPV1 on day 10 (Figure 4C). No signal was detected in the smooth muscle cells of the blood vessels for this receptor. Circular and longitudinal muscle layers presented an intense signal for TRPV1 on day 8 and 10 (Figure 3F).

### Spatiotemporal expression of CB1, CB2 and TRPV1 receptor - Days 12 to 14 of pregnancy

**Figure 5:**
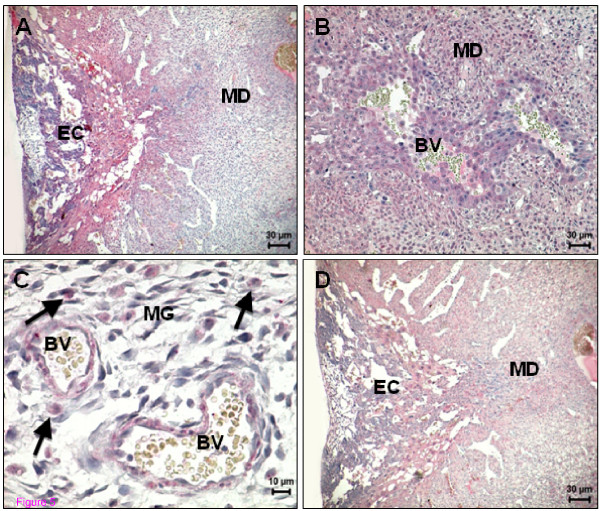
**CB1 and TRPV1 immunoreactivity on day 12 of pregnancy**. (A) CB1 expression in decidualized cells in the mesometrial region; note that positive staining was restricted to the edges of mesometrial decidua nearest to the ectoplacental cone, which also is positive for CB1; (B) Expression of CB1 in the endovascular trophoblast cells; (C) Expression of CB1 in the metrial gland. Smooth muscle cells of blood vessels and uNK cells (arrows) are positive; (D) TRPV1 expression in decidualized cells in the mesometrial region in the vicinity of ectoplacental cone, which show some positivity. BV - Blood vessel; EC - Ectoplacental cone; MD - Mesometrial decidua; MG - Metrial gland.

On day 12, the decidual cells near to the early placenta called the ectoplacental cone, showed immunopositivity for CB1 (Figure 5A). CB2 was also present, but expressed with a lower intensity, though with the same pattern to that of CB1. On day 12, CB1 was first detected in the trophoblast cells of the ectoplacental cone (Figure 5A) and in the endovascular trophoblast cells (Figure 5B). Immunostaining for CB1 was detected in the smooth muscle cells of the blood vessels (Figure 5C). Immunopositive staining for both receptors was observed in the metrial gland, localized to uNK cells (Figure 5C).

**Figure 6:**
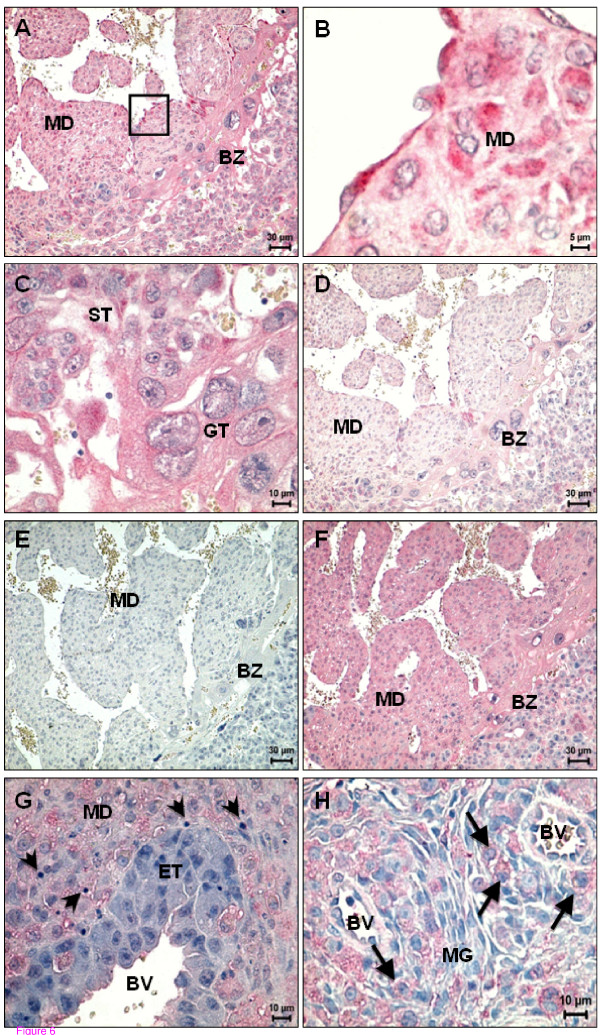
**Spatial distribution of cannabinoid and vanilloid receptors on day 14 of pregnancy**. (A) CB1 expression, in the mesometrial decidua and basal zone of placenta; (B) High magnification of the square depicted in (A); note the positive decidual and endothelial cells. (C) Higher magnification image of the basal zone. The giant trophoblast and spongiotrophoblast cells are positive for CB1; (D) Expression of CB2 is lower than CB1 in the mesometrial decidua and basal zone; (E) Negative control for TRPV1; (F) TRPV1 expression in the mesometrial decidua and basal zone; (G) High magnification of the mesometrial decidua and endovascular trophoblast cells; note the lack of staining for TRPV1 in these trophoblast cells and the presence of apoptotic bodies in the decidua (arrowheads); (H) Expression of TRPV1 is particularly evident in uNK cells in the metrial gland region (arrows). BV - Blood vessel; BZ - Basal zone; ET - Endovascular trophoblast cells; GT - Giant trophoblast cells; MD - Mesometrial decidua; MG - Metrial gland; ST - Spongiotrophoblast cells.

On day 14, the expression of immunoreactive CB1 and CB2 was found in all mesometrial decidual cells and in the placenta (giant trophoblast and spongiotrophoblastic cells) (Figure 6A, C, D). On this day, the endothelial cells were also positive for CB1 (Figure 6A, B).

TRPV1 immunoreactivity in the mesometrial decidua increased in intensity in the decidual cells and in uNK cells from days 12 to 14 (Figure 5D, 6F). There was strong immunoreactivity for this receptor in the uNK cells of the metrial gland (Figure 6G, H). Contrary to what was found for CB1, the endovascular trophoblast cells were negative for TRPV1 (Figure 6G). A very weak signal was observed in the circular compared to the longitudinal smooth muscle layer. Within placental cells, TRPV1 immunoreactivity was confined to the giant cells and to a few spongiotrophoblast cells (Figure 6F).

### Spatiotemporal expression of CB1, CB2 and TRPV1 receptor protein - Days 16 to 19 of pregnancy

**Figure 7:**
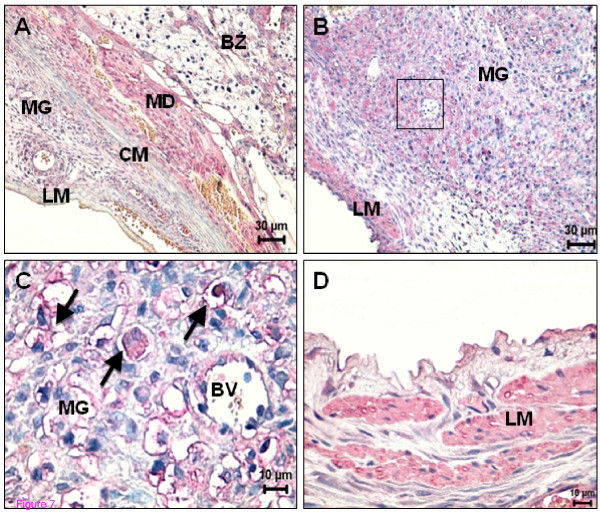
**Spatial distribution of CB1 and TRPV1 in the uterus on day 16 and 19 of pregnancy**. (A) On day 16, decidual and trophoblast cells from the basal zone are positive for CB1. Note the lack of expression in circular and longitudinal muscle layer; (B) In the metrial gland, the expression of TRPV1 at day 19 is mainly localized in uNK cells and in longitudinal muscle layer. (C) High power of the square depicted in (B). Expression of TRPV1 is particularly evident in uNK cells (arrows). (D) High power showing an intense positive signal for TRPV1 in the longitudinal muscle layer. BV - Blood vessel; BZ - Basal zone; CM- Circular muscle layer; LM - Longitudinal muscle layer; MD - Mesometrial decidua; MG - Metrial gland.

Expression of CB1 was predominantly localized in the regressing mesometrial decidua (Figure 7A) and continued to be expressed in the placenta. On day 19, the expression of both cannabinoid receptors were unchanged compared to day 16. On day 19, TRPV1 continued to be expressed in the uNK cells and also in the longitudinal smooth muscle layer (Figure 7B, C, D).

## Discussion

Although the endocannabinoid system has been investigated at the time of implantation in the endometrium of the mouse model [21], there are to our knowledge no studies that have investigated this system in the post-implantation period. The widespread expression of cannabinoid receptors in numerous organs suggested that endocannabinoids might have functions other than psychoactive. For example, evidence has emerged to support a role for the endocannabinoid system in blastocyst development and implantation following the localisation of the cannabinoid receptors, CB1 and CB2, to the blastocyst and to the uterus during the peri-implantation period [20,32,33].

We used Q-PCR and western blot to demonstrate transcript and protein levels throughout pregnancy for the anandamide-binding receptors CB1, CB2 and TRPV1, and uterine tissue, distribution of these receptors was revealed by immunohistochemistry.

In the rat and other mammals, including the human, fibroblast-like endometrial stromal cells undergo synchronized proliferation and differentiation in response to the implanting blastocyst, a phenomenon designated as decidualization [24]. In the rat, after proliferation, the decidual tissue goes through a cycle of regression, which begins at day 10 in the antimesometrium side and continues, in the mesometrium pole, until the end of pregnancy [26,27]. This biological programming of endometrial stromal fibroblasts into decidual cells is thought to be affected by locally released factors produced by maternal tissues and blastocyst [31,34,35]. Though the exact mechanisms controlling this process in normal pregnancy are unknown, they may be related to the endocannabinoid system [36].

Immunohistochemistry revealed that CB1 is expressed mainly in decidual cells throughout gestation suggesting that an action mediated by CB1 is not only associated with implantation [19], but also with the maintenance of pregnancy. Our western blot results showed that CB1 was upregulated during midpregnancy, being ~1.5-fold higher on day 12, which is not in accordance with Q-PCR data, suggesting that the expression of proteins is transcription-unregulated. In fact, the uterine remodelling is a dynamic and highly regulated process associated with the synthesis of different types of molecules, like growth factors and hormones. These molecules may be critical for the regulation of the translational process, what explain the differences observed between Q-PCR and western blot results. After day 12, when maximum development of the mesometrium decidua occurs, the decidualization process is completed and the mesometrial decidual cells undergo apoptosis [27]. By contrast, by day 12 of gestation the antimesometrium has already undergone regression.

During the period up to day 10 the fibroblast stromal cells of this region proliferate and differentiate, whereas by day 12 only a few cells remain. Indeed, the expression levels of CB1 is higher on the antimesometrium side on day 10 suggesting that CB1 may be involved in the programmed cell death that occurs in this period in the antimesometrium. Moreover, Kessler *et al*. showed that a CB1 agonist inhibits human decidualization and stimulates apoptosis by a cAMP-dependent mechanism [36] indicating that anandamide signals or initiates apoptosis. In addition, we have shown the involvement of anandamide in mesometrial decidual cell death through CB1, suggesting a role for this receptor in decidual regression [28].

Although CB2 was first isolated from immune cells with particularly high levels in B lymphocytes and natural killer cells [37], little is known about the physiological role of this receptor. In this work, CB2 presented a similar expression pattern to CB1, though with less intensity and without significant differences throughout pregnancy as revealed by western blot analysis. It is possible that CB2 might contribute to the control of cytokine networks responsible for maternal/fetal cross talk.

Buckley *et al*. described CB1 and CB2 receptor mRNAs in the outer longitudinal layer and in the inner circular layer of myometrium of the pregnant rat uterus [38]. However, it was detected immunoreactivity for all the receptors in the circular muscle layer, with decreasing intensity as gestation advances. This raises the possibility that these receptors may play a role in myometrial contractility, though a direct effect of AEA and THC as relaxants on human myometrium contractility *in vitro*, mediated through CB1, has been described [39]. Conversely, we also detected a strong reactivity for the vanilloid receptor in the longitudinal muscle layer throughout gestation. These data demonstrate differential regulation of TRPV1 expression between the muscle layers that would result in enhancement of relaxation activity in the longitudinal muscle of the gravid horn during pregnancy, necessary to achieve embryo development and parturition. In the human, a dramatic increase in plasma anandamide levels during labour at term compared with non-labouring women has been described [15] suggesting a role for anandamide in labour. Thus, we hypothesise that TRPV1 activation mediated by anandamide might contribute to the ability of the outer myometrial layer to generate optimal contractile activity during labour. In the same way, AEA may induce vasodilatation in vascular tissues, which appears to be mediated through the CB1 receptor [40,41]. Our study also demonstrated that cannabinoid receptor expression, mainly CB1, in the smooth muscle cells of blood vessels occurs until day 12 of pregnancy, suggesting that anandamide, acting through the vascular CB1, may be involved in the vasodilatation which is crucial for the development of a normal pregnancy. In addition, anandamide produces vasorelaxation in different vascular beds in an endothelium-dependent and endothelium-independent manner [10]. We found expression of CB1 in endothelial cells from day 12 until the end of pregnancy and although anandamide has been shown to exert some of its effects directly on vascular smooth muscle via the CB1 receptor [42], a direct action of anandamide on the endothelium is also possible.

The presence of immunoreactive TRPV1 in uNK cells was observed from day 10 until degranulation, contrary to CB1 expression that diminishes around day 12. The distinctive expression of TRPV1 and the loss of CB1 expression could be related to the induction of the apoptosis that occurs in these cells during this period [27], as has been suggested for other cell types [43].

In human pregnancy, cannabinoid receptor expression has been detected in first trimester placental tissues [16,44] and at term [33]. Our results also revealed expression of CB1 in the rat endovascular trophoblast, giant trophoblast and spongiotrophoblast cells, suggesting that CB1 might be involved in trophoblasts-associated uterine remodelling mechanisms. Moreover, as TRPV1 was primarily found in giant trophoblast cells, activation of this receptor may counter-balance the trophoblast invasion.

## Conclusion

All these data indicate that a tightly regulated level of endocannabinoids is crucial during placental development. The differential spatio-temporal expression patterns of the cannabinoid and vanilloid receptors suggest that there is an uncoupling of these two types of anandamide-binding receptors in mid/late gestation that are related to changes in cell phenotype. The reasons for this are not clear, but it may be explained by a differential role for anandamide in the various cell types, which may also be dose-dependent. To our knowledge, this is the first study providing direct evidence of the existence of anandamide-binding receptors in uterine tissues throughout pregnancy and for supporting a role for the endocannabinoid system during the period of placental development.

## Competing interests

The authors declare that they have no competing interests.

## Authors' contributions

BMF carried out all the experiments and drafted the manuscript. AHT designed the primers and participated in the correction of the manuscript. JCK participated in its correction and SCB in its design. GCS and NAT conceived and coordinated the study and helped to draft the manuscript. All the authors read and approved the final manuscript.
